# Assessing the implementation of COVID-19 structured reporting templates for chest radiography: a scoping review

**DOI:** 10.1259/bjro.20220058

**Published:** 2023-03-28

**Authors:** Peter A O'Reilly, Sarah Lewis, Warren Reed

**Affiliations:** 1 Academic, Discipline of Medical Imaging Science, The University of Sydney School of Health Sciences, Camperdown, Sydney, Australia; 2 Associate Dean Research Performance, Faculty of Medicine and Health, The University of Sydney School of Health Sciences, Camperdown, Sydney, Australia; 3 Program Director, Bachelor of Applied Science (Diagnostic Radiography), The University of Sydney School of Health Sciences, Camperdown, Sydney, Australia

## Abstract

**Objective:**

One of the common modalities used in imaging COVID-19 positive patients is chest radiography (CXR), and serves as a valuable imaging method to diagnose and monitor a patients’ condition. Structured reporting templates are regularly used for the assessment of COVID-19 CXRs and are supported by international radiological societies. This review has investigated the use of structured templates for reporting COVID-19 CXRs.

**Methods:**

A scoping review was conducted on literature published between 2020 and 2022 using Medline, Embase, Scopus, Web of Science, and manual searches. An essential criterion for the inclusion of the articles was the use of reporting methods employing either a structured quantitative or qualitative reporting method. Thematic analyses of both reporting designs were then undertaken to evaluate utility and implementation.

**Results:**

Fifty articles were found with the quantitative reporting method used in 47 articles whilst 3 articles were found employing a qualitative design. Two quantitative reporting tools (Brixia and RALE) were used in 33 studies, with other studies using variations of these methods. Brixia and RALE both use a posteroanterior or supine CXR divided into sections, Brixia with six and RALE with four sections. Each section is scaled numerically depending on the level of infection. The qualitative templates relied on selecting the best descriptor of the presence of COVID-19 radiological appearances. Grey literature from 10 international professional radiology societies were also included in this review. The majority of the radiology societies recommend a qualitative template for reporting COVID-19 CXRs.

**Conclusion:**

Most studies employed quantitative reporting methods which contrasted with the structured qualitative reporting template advocated by most radiological societies. The reasons for this are not entirely clear. There is also a lack of research literature on both the implementation of the templates or comparing both template types, indicating that the use of structured radiology reporting types may be an underdeveloped clinical strategy and research methodology.

**Advances in knowledge:**

This scoping review is unique in that it has undertaken an examination of the utility of the quantitative and qualitative structured reporting templates for COVID-19 CXRs. Moreover, through this review, the material examined has allowed a comparison of both instruments, clearly showing the favoured style of structured reporting by clinicians. At the time of the database interrogation, there were no studies found had undertaken such examinations of both reporting instruments. Moreover, due to the enduring influence of COVID-19 on global health, this scoping review is timely in examining the most innovative structured reporting tools that could be used in the reporting of COVID-19 CXRs. This report could assist clinicians in decision-making regarding templated COVID-19 reports.

## Introduction

On 31 December 2019, the first report of a new respiratory virus emerged from Wuhan, China, where 27 patients were diagnosed with what was initially described as a novel coronavirus.^1^ The chest radiographs (CXRs) of a number of these patients identified pneumonias exhibiting ground-glass opacities (GGOs).^1^ Within early February 2020, the World Health Organisation (WHO) had named the disease as COVID-19, and the virus as a severe acute respiratory syndrome coronavirus (SARS-CoV-2).^2^ Additionally, by March 2020, the WHO declared that COVID-19 was a pandemic and stated it was incumbent on all nations to undertake mitigation strategies via key approaches—to detect, protect and treat against the virus and through this, to innovate and learn.^3^

From a radiological perspective, COVID-19 infiltrates the nasopharynx via droplet formation and can quickly enter the lower respiratory system.^4^ Once within the lungs, COVID-19 subsequently features as air space opacities such as GGO, consolidation and reticular interstitial thickening, which is referred to at times as crazy paving.^4^ CT has a higher sensitivity for the detection of GGO^5,6^ but CXRs are useful for the ongoing clinical management of the COVID-19 patient and have clear advantages over CT with respect to more rapid imaging results,^7^ availability at the point of care^8,9^ and offer a more affordable and available option than CT, especially in developing countries.^10,11^ GGO is defined as increased pulmonary opacity without obscuring the underlying margins of bronchi and vascular margins, whereas consolidation obscures the appearance of the bronchial and vascular margins.^4^ The location of infection is another important consideration as COVID-19 is typically seen in multifocal locations predominantly in bilateral peripheral and basal aspects of the chest.^4^

Many international radiology associations responded to the challenges of COVID-19 by developing and recommending specific CXR reporting tools that utilise a structured template that could facilitate reporting in a more standardised way other than traditional free text methods.^12–15^ There are several advocated benefits that reporting templates can offer^16^ including a uniform description of COVID-19 infection, a choice of language that is readily understood by a range of referrers, and the use of less ambiguous descriptions.^17–19^ Other advantages are the ease of use with templates organised into subheadings relating to disease or an organ system formatted by the selection from bulleted lists or check boxes.^20,21^

The purpose of this scoping review was to investigate how such structured reporting templates have been used, or recommended to be used, in the reporting of CXRs in patients with, or suspected of having COVID-19. One of the main inclusion criteria employed for the article selection was in the use of either a qualitative or quantitative design of the reporting template. The selected articles were also examined to discover which conclusions were found in the use of either reporting design to justify their use as a methodology or outcome for patient diagnosis and/or patient care. Further, this review also sought to highlight any gaps that may exist in the literature regarding the use of these structured reporting instruments that may inform future research.

## Methods

A scoping review was conducted to evaluate literature published on the application of structured reporting tools used in the reporting of COVID-19 CXRs. The review was based on the original framework proposed by Arksey and O’Malley.^22^

### Identifying the research question

The key research questions employed in this scoping review are as follows:

- What is the extent in the literature on the use or utility of templates for reporting on patients with suspected or confirmed COVID-19 disease using CXR?
- What gaps exist in the literature concerning the use of either quantitative or qualitative methods including the research on efficacy or comparison of both reporting techniques.

### Identifying the relevant studies

An experienced health librarian assisted in the initial selection of these key broad terms as the starting method of our database interrogation and in the selection of the four most appropriate medical imaging databases to use in the study. We examined original, peer-reviewed articles, and grey literature (professional/national guidelines for clinical practice) published in English between January 2020 until April 2022. Systematic searches were undertaken through the four main medical imaging electronic bibliographic databases of Medline, Scopus, Web of Science and Embase. In conjunction with the electronic searches, periodic hand searches using Google Scholar and the online resources offered by the University of Sydney library were used. The key search words, Boolean operators and search tools are summarised in Table 1.

**Table 1.** Search words and search tools

KEY SEARCH WORDS	BOOLEAN OPERATORS	SPECIFIC SEARCH TOOLS
COVID-19		-
COVID*.mp	-	.mp *
Quantitative.mp	-	*
Corona?virus infections/	or	? * .mp
Coronavirus infection*.mp	-	* .mp
one or two or three or four or 5	or and	all used
Chest X-ray*.mp	-	* .mp
Chest X-ray	or	-
Mobile chest X-ray*.mp	-	.mp
Emergency radiograph*.mp	-	.mp *
ICU chest X-ray*.mp	or	.mp *
Intensive care chest X-ray*.mp	-	.mp *
7 or 8 or 9 or 10 or 11	or	all tools used
Chest severity score in COVID-19	-	-
Emergency mobile X-ray.mp	or and	-
Various other combinations	or and	? .mp *.

ICU, intensive care unit.

Table 2 is a summary of the initial Medline search where all relevant clinical search terms were employed. Giving substantial initial results, these terms were then applied to the Embase, Web of Science and Scopus databases. Table 3 represents the total of all studies before exclusion and inclusion criteria were applied. In addition to the 77 articles found electronically, there were an additional 14 articles retrieved manually through Google Scholar. Another 10 studies from international radiologic societies examining structured reporting templates (grey literature) were added to the list.

**Table 2.** Medline search results

SELECTION	TERMS	RESULTS
1	COVID-19	104,070
2	Covid*.mp	170,472
3	Quantitative.mp	105,052
4	Corona?virus infections/	45,090
5	Coronavirus infection*.mp	188,505
6	1 or 2 or 3 or 4 or 5	26,130
7	Chest X-ray*.mp	22,318
8	Chest radiograph*.mp	10
9	Mobile chest X-ray*.mp	4
10	ICU chest X-ray*.mp	3
11	Intensive care chest X-ray*.mp	47,105
12	7 or 8 or 9 or 10 or 11	1,413
13	Chest severity score in COVID-19	2
14	Emergency mobile X-ray.mp	2
15–75	Various other combinations	21
	TOTAL	21

ICU, intensive care unit.

**Table 3.** Database results

SEARCH ENGINE	PLATFORM	TOTAL SEARCHES	RESULTS
Medline	Ovid SP	75	21
Embase	Ovid SP	81	32
Web of Science	Clarivate	62	20
Scopus	Elsevier	47	4
Total Studies Found 77			
& Utilizing Google scholar (*n* = 14) 91 & Including grey literature (*n* = 10). 101			

### Study selection criteria

#### Research articles

Research articles needed to satisfy a range of criteria to be included in this review. The reporting template needed to be a format that was a pre-designed document with pre-set options. Moreover, the reporting language type employed in the pre-designed document needed to utilise either a qualitative or quantitative description of disease. Publications earlier than January 2020, articles with no evidence of peer review (except national guidelines that subsequently were updated to include COVID-19) and variations on the theme of machine learning where the outcome was to describe a machine learning process rather than a clinical outcome or utility, were all excluded. Table 4 outlines both the key inclusion as well as exclusion criteria.

**Table 4.** Inclusion and exclusion criteria

CRITERION	INCLUSION	EXCLUSION
Time period	2020 to 2022	Studies from and earlier than 2020
Type of article	Original research	Not original research
Grey literature	Radiological societies	Non-society statements
Peer reviewed	Yes	No stated peer review
Language	English	Non-English studies
Population	COVID-19	Other respiratory foci
Infection	SARS-CoV-2	Any other infectious agent
Examination	CXR	CT chest or other imaging forms
Report design	Template	Non-template designs
Report design	Structured	Standard free text reporting methods
Study method /design	Quantitative	Machine learning, neural networks
Study method /design	Qualitative	Deep learning, artificial intelligence
Study method/design	-	Learning algorithms

CXR, Chest X-ray.

### Charting the data

Data extraction tables were generated and identified the following information: authors, publication date, theme of the study or study purpose, reporting methods, structured reporting of either qualitative or quantitative designs. In addition to the studies that satisfied inclusion criteria, grey literature was included as it yielded important data for later analysis.

#### Charted data

**Figure 1.**
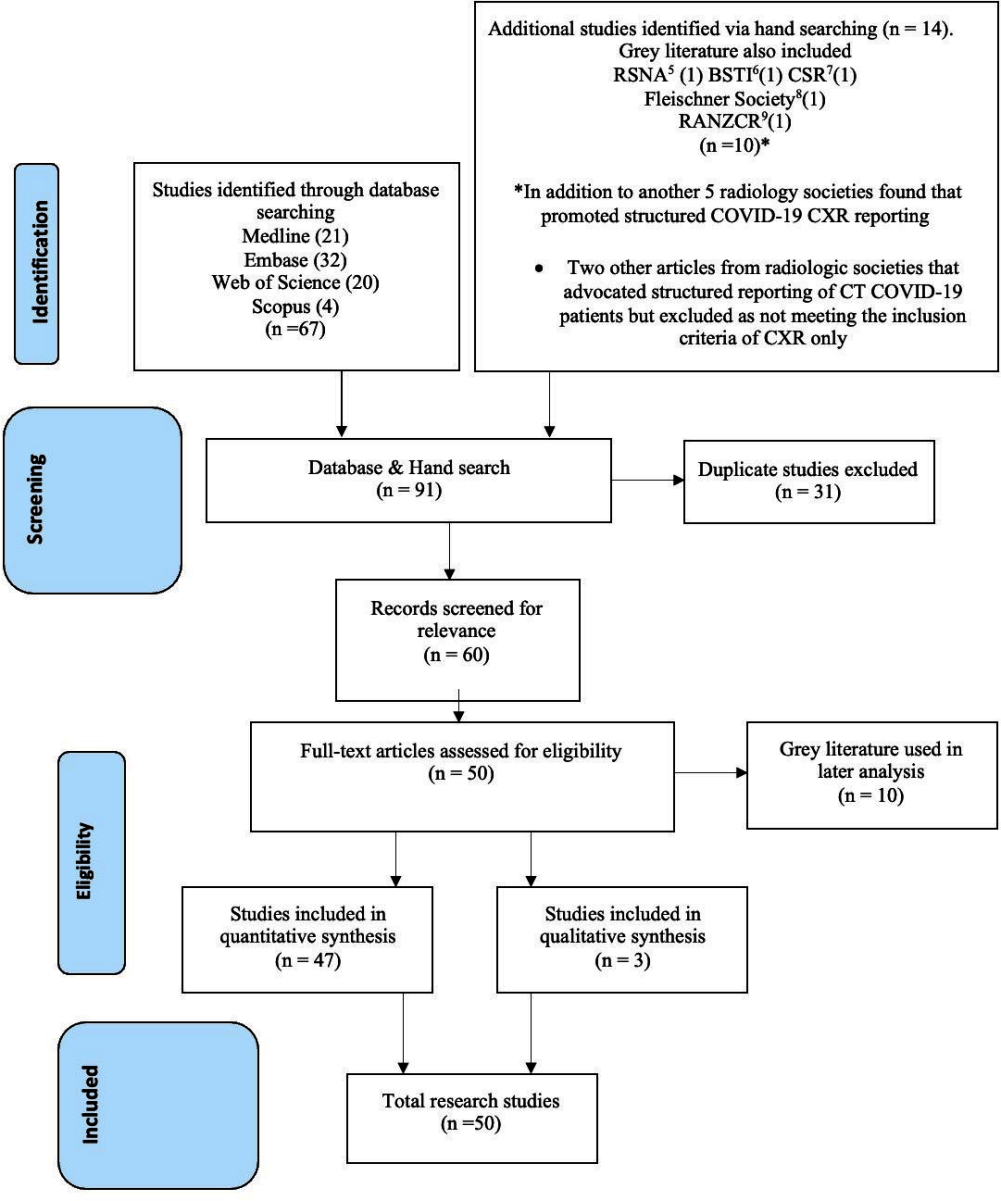
presents the PRISMA diagram of the literature search with 50 papers included in the synthesis.

Figure 1 PRISMA 2020 Flow diagram for final article selection

## Results

There were 50 articles examined after inclusion and exclusion criteria were applied. 10 radiological societies’ recommendations in addition to the 50 articles retrieved were also included for analysis. Of the 50 articles, 47 employed a quantitative design whilst the remaining 3 articles used a qualitative method. The results that follow are headed Radiological Societies, Qualitative Structured Reports and Quantitative Structured reports.

### Radiological societies (Grey literature)

The criteria for inclusion or exclusion of grey literature underwent the same process that applied to all material found via the web search process (outlined in Table 4). As a part of the literature search, 10 radiological societies were found that advocated structured reporting of COVID-19 CXRs. Of these, seven recommended a qualitative design, whereas the other three recommended a quantitative design. Table 5 illustrates all available national/professional radiology societies found that have listed a recommended structured template for COVID-19 imaging, using either the qualitative or quantitative method.

**Table 5.** Ten Radiological societies’ recommended reporting tools for COVID-19 CXRs

SOURCE	TYPE	FINDINGS
The Royal Australian and New Zealand College of Radiologists (RANZCR)	Qualitative	Findings assigned to one of four categories: Normal Indeterminate Typical Other diagnosis favoured
Sri Lanka Journal of Radiology (SLJR): Abeywardana et. al.	Quantitative	Each lung is divided into four zones and a score of 0 or 1 is assigned to each zone based on presence or absence, respectively, of GGOs or consolidation. A total severity score (out of 8) is calculated by summing the scores of all zones.
British Society of Thoracic Medicine (BSTI).Types: Baseline template ICU template Follow up template	Qualitative	Findings assigned to one of four categories: Normal Classic: probable for COVID-19 Indeterminate for COVID-19 Non-COVID −19
European Society of Radiology: Yates et.al.	Qualitative	Findings assigned to one of five categories: Characteristic High suspicion Indeterminate Unlikely Normal
Canadian Society of Thoracic Radiology	Qualitative	Findings assigned to one of three categories: Typical appearance (of COVID-19) Non-specific appearance (of COVID-19) Negative pneumonia
Russian Society of Roentgenologists and Radiologists	Qualitative	Findings assigned to one of three categories: High probability of COVID-19 pneumonia Medium probability of COVID-19 pneumonia Low probability of COVID-19 pneumonia.
Radiological Society of North America Wong et al	Quantitative	A score of 0–4 is assigned to each lung based on extent of consolidation or GGOs. A total severity score (out of 8) is calculated by summing the scores of each lung.
Korean Journal of Radiology (KJR): Jung et. al.	Quantitative	Each lung is divided into four zones A score of 0–4 is assigned to each zone based on extent of pneumonia. A total severity score (out of 24) is calculated by summing the scores of all zones
Guidelines of the University of Pennsylvania (Endorsed by the RSNA)	Qualitative	Findings assigned to one of four categories: No Radiographic evidence Indeterminate COVID-19 other types of pneumonia Focal pneumonia Bilateral multifocal COVID-19 pneumonia
Radiological Society of North America (Radreport.org)	Qualitative	Findings assigned to one of five categories: Normal or non-infectious Features compatible with COVID-19 or other infections Suspicious for COVID-19 Typical for COVID-19
TOTAL 10 Qualitative (7) Quantitative (3)		

CXR, chest X-ray; GGO, ground-glass opacity.

### Qualitative structured reports

The intent of qualitative structured reports is to provide classification or descriptors that closely relate to the presence or absence of radiological signs of COVID-19 or to assign an alternative diagnosis. Structured reporting templates often list an agreed lexicon, as generated by professional societies or colleges, and generally do not require the radiologists to alter these descriptors but rather use standardised language. For COVID-19 structured reporting, it appears the intent is to provide a rapid assignment of classification of disease presence, and in some cases severity, although there is limited research on the construction, validity and implementation of these qualitative templates.

The first of the three qualitative studies included in this review, that of Borakati et al^23^ adopted the British Society of Thoracic Imaging (BSTI) guidelines^24^ and sought to determine the diagnostic accuracy of COVID-19 CXRs compared to chest CT using the BSTI template (Appendix A) in examining the health outcomes of their patient cohort. The study was undertaken in a British hospital emergency department and the results of 1198 eligible patients showed that the COVID-19 CXRs (0.56) revealed a poor sensitivity in the detection of COVID-19 compared to CT (0.85, significantly higher). There were several described limitations in the research such as the non-inclusion of relevant co-variants of blood markers of infection status that were available in all patients’ files. Further, there was a significant amount of missing clinical data in many patients’ records. Moreover, there was only one reporting radiologist which excluded any inter-rater reliability and a potential for undetected under- or over-reporting of COVID-19. The authors conclusions specifically stated that the use of the CXR to assess COVID-19 status was not statistically correlated with any clinical findings, which included “vital signs, laboratory parameters or 30 day outcomes”. There was however a strong correlation found between radiology findings on CT compared to clinical status.^23^

The second, a study by Durrani et al^25^ also used the BSTI template guidelines (Appendix A) to study its usefulness in a different international cohort of 30 COVID-19 positive patients. Each patient over a 20-day period underwent portable CXRs and were then classified using the descriptions that fitted the radiological appearances of the BSTI guidelines. The study found that the BSTI descriptors matched their cohort, but suggested an improvement in the descriptors of classic COVID-19 with the inclusion of mid-zonal involvement. The authors hoped the study could serve as a benchmark examination that similar health environments with X-ray facilities could copy as an affordable tool for the assessment of COVID-19 infected patients. There were important study limitations listed by the authors. The cohort size was only 30 patients and there was no access to serial imaging to inspect progression of the disease or to examine variable appearances in any of the patients. Beyond the author’s stated limitations, there were no details given of the number of reporting radiologists or any available statistical data to assess radiologist concordance.^25^

The third qualitative article found in this review was that of Yates et al^26^ who sought to examine the utility of a self-designed reporting template on patients admitted in an emergency department who had a suspected SARS-CoV-2 infection. These results were compared to the RT-PCR test, the gold-standard in assessing SARS-CoV-2 infection. There were two radiologists reviewing all CXRs and blind to the RT-PCR results. The authors employed a self-developed qualitative reporting template defined by using five levels of disease: ‘characteristic pattern’, ‘high suspicion pattern’, ‘indeterminate pattern’, ‘unlikely pattern’ and a ‘normal pattern’ (Appendix B). There were 582 patients’ CXRs reported and the absolute concordance between the two radiologists was 71% (414/582). There was also a Fleiss-Cohen weighted Cohen’s k of 81 (95% confidence interval, 0.78–0.85). They found that a positive COVID-19 CXR was predictive of a positive RT-PCR and chest radiography can be useful in recognising undiagnosed COVID-19.^26^ The reporting template designed by the authors was described as “proof of concept” and the feasibility of the reporting tool, as stated by the authors, should be verified by its use in other radiology facilities, examining other cohorts of COVID-19 patients.^26^

**Figure 2.**
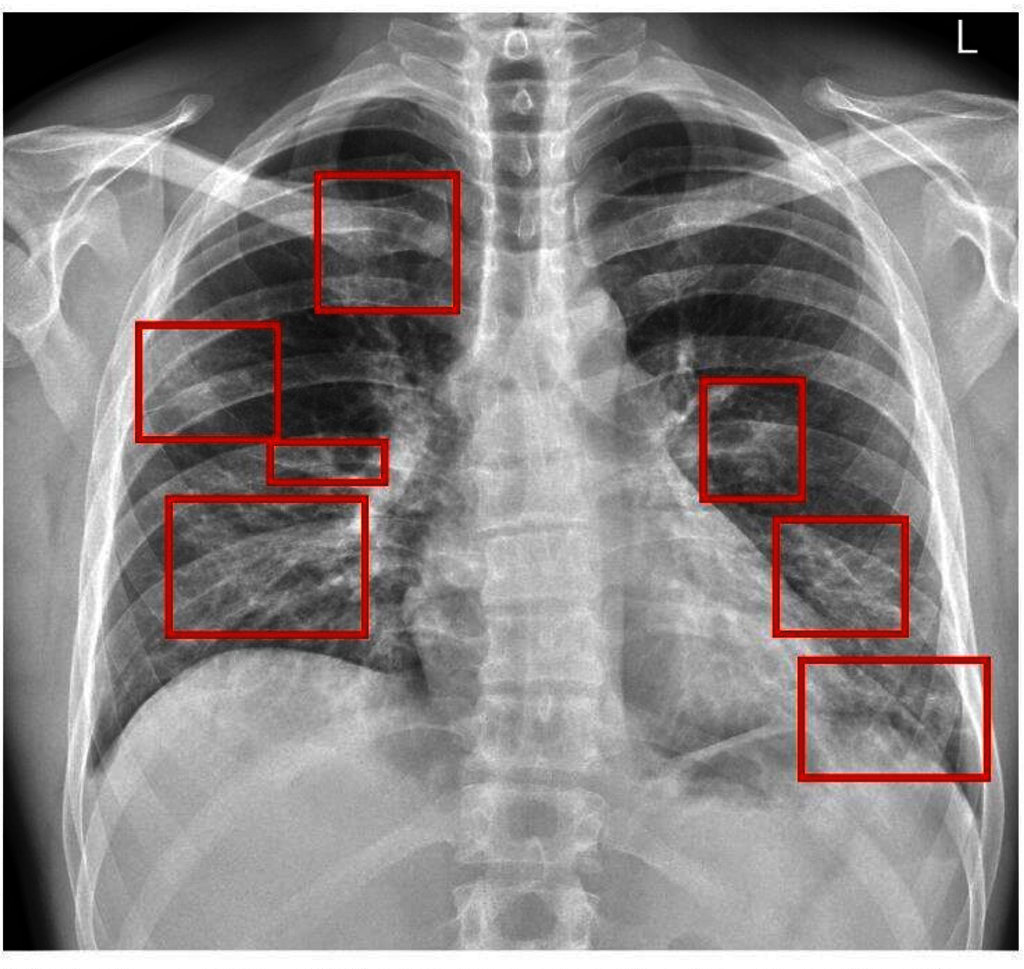
shows areas of infection in varying size, density and location.

Figure 2 An example of the qualitative method of CXR assessment.

The qualitative design typically use terms that define the characteristic appearance of COVID-19 infections: consolidation, GGO and crazy paving. Moreover, location is important as COVID-19 is typically seen in bilateral peripheral and basal multifocal locations. The reports also focus on overall descriptions employing terms such as characteristic pattern, high suspicion, likely pattern, indeterminate and classic/probable. One advantage of the qualitative assessment tool is that if there is an agreed lexicon by clinical radiology, then the reporting Radiologist can use the terms confidently and the descriptors in time become more commonly used. There is also a prompt report turn-around which is characteristic of all templated reporting instruments. The major disadvantage is that these terms can be seen by the referrer, who is not familiar with agreed lexicons, as vague, too descriptive and equivocal.

### Quantitative structured reports

Quantitative structured reporting instruments examined in this review used numerical data or disease descriptions associated with numerical data to describe levels of COVID-19 disease by segmenting the lung fields into regions, lobes, or quadrants and assigning a score or severity index to show the extent of disease. The Brixia^27^ and Radiographic Assessment of Lung Oedema (RALE)^28^ reporting tools are by far the most common instruments used of the 47 quantitative studies found, and were the reporting tool of choice in 33 studies—70% of all quantitative studies examined.

The use of the Brixia scoring system, the most adopted quantitative scoring method used in 40% of all quantitative reporting studies found in this review, was first designed and used in early 2020 where concentrated areas of SARS-CoV-2 outbreaks were initially observed in Italy.^27^ The study was undertaken in the Italian city of Brescia (Roman description as Brixia), hence the name. This was one of the earliest published studies examining the prognostic value of using a quantitative scoring system in the assessment of the CXRs of COVID-19 patients.^27^ The study employed a numerical grading system by way of an 18-point severity scale that applied to a front-on CXR divided anatomically evenly into six regions. The scoring ranged from 0 with no lung abnormalities, a score of 1 which signified interstitial infiltrates, a score of 2 which signified interstitial and alveolar infiltrates and the highest score of 3 which determined alveolar predominance. Hence, the worst infectious state for a patient would be a totalled six regions scoring alveolar predominance in each, giving a final score of 18.^27^ The findings in this study found the Brixia scoring method was an effective tool to monitor the progression of COVID-19 infection, particularly in intensive care.^27^

The second most common quantitative structured reporting tool, which comprised 30% of all quantitative studies found in this review, is known as RALE and was initially derived from an assessment tool of pulmonary oedema.^28^ The application of RALE to the COVID-19 CXRs is more complex than Brixia. RALE divides the anteroposterior or posterioanterior CXR into four quadrants and uses numerical scores of consolidation (1–4) and density (1–3) x four quadrants. The consolidation in each segment refers to the extent or the percentage of the alveolar opacification in that segment. The density score rates the overall density of that opacification. The density criteria of 1 signified hazy, criteria 2 signified moderate and 3 signified dense. As a result, RALE examines the extent as well of intensity (density) of infection. An example in the use of RALE in a worst-case scenario is outlined in Appendix C.

The use of the RALE or Brixia systems and other author-constructed quantitative reporting templates are based on three important propositions that underpin all quantitative reporting tools found in this scoping review. Firstly, does the reporting tool have utility, secondly does the reporting tool have a predictive value, and finally does the reporting tool act in a manner to verify other clinical findings and in so doing, act as an independent variable.

There were numerous studies in this review that verified the utility of the quantitative reporting tool. For example, in the study by Al-Yousif et al,^29^ 415 known COVID-19 patients were examined by use of the RALE reporting tool. The RALE score was found to be efficacious in assessing the degree of COVID-19 infection and assisted treatment management. Another study examining the efficacy of the RALE reporting tool was Orsi et al^30^ who examined 155 COVID-19 positive patients. The study conclusion stated that the visual scoring on each CXR was easy and highly reproducible and reflected the clinical severity, assisting patient treatment. The study by Abo-Hedibah et al^31^ employed the Brixia method in the examination 325 COVID-19 positive cases. The study concluded by stating that the use of Brixia in the assessment of 325 patients was a reliable method to assess the severity of pulmonary parenchymal disease. They also stated that this reporting tool was particularly accurate in assessing moderate to severe cases of COVID −19.^31^ An extensive list of studies that sought to verify the utility of the quantitative reporting tool are found in Appendix D.

The second feature, the prognostic value of the structured quantitative report, was found in many studies and served as a useful tool to predict patient outcomes. For example, Balbi et al^18^ examined the initial CXR of 340 COVID-19 patients admitted to an emergency setting using the Brixia reporting template to determine if the CXR could be an early predictor of mortality. The study conclusion was that the reporting template was a predictive tool that can identify patients at risk of death as well as the need for ventilatory support. Yasin and Gouda^32^ similarly sought to identify the prognostic value of their reporting tool. Their instrument divided each lung into two regions and then four scores applied to each region. A score of 1 equated to ≤25% of infection; a score of 2 equated to 25–50% of infection; a score of 3 equated to 50–75% and a score of 4 equated to >75% of infection. The highest level of infection, >75% of infection in both lungs, scored 8. They found that the reporting instrument was an accurate method of predicting the future severity of SARS-CoV-2 infection. Iftikhar et al^33^ reviewed 200 diagnosed COVID-19 patients. The clinical rationale for this study was to determine if the quantitative reporting tool was a predictive risk for hospital admission and eventual intubation/mechanical ventilation hospital disease progressed in time, and if this progression correlated with intubation and risk of death. The study found that the severity score did have predictive value in patient admission and later intubation. An extensive list of studies utilising the predictive method are found in Appendix E.

Several quantitative studies used either Brixia or RALE reporting tool as the independent variable in examining the relationship between COVID-19 CXRs and variables of health status. An example is the study by Basilico et al^34^ which examined the relationship between rates of COVID-19 infection and adiposity indexes in a cohort of 215 patients. The indexes used were abdominal obesity and body mass index (BMI). The results indicated that increased abdominal obesity levels correlated with a higher rate of COVID-19 infection than those patients with elevated BMI.^34^ A study by Rejeki et al^35^ looked at the use of plasma convalescent transfusion in reducing COVID-19 infection and found that the Brixia scoring method in conjunction with other laboratory parameters were able to determine the efficacy of the plasma treatment.^35^ Christanto et al^36^ sought to determine the relationship between diabetes mellitus and COVID-19 in a cohort of 538 patients with both pathologies. Both RALE and Brixia reporting tools were used and found a substantial association between increased hyperglycaemia and lung severity in COVID-19 patients aged 60 years or older.^3^

**Figure 3.**
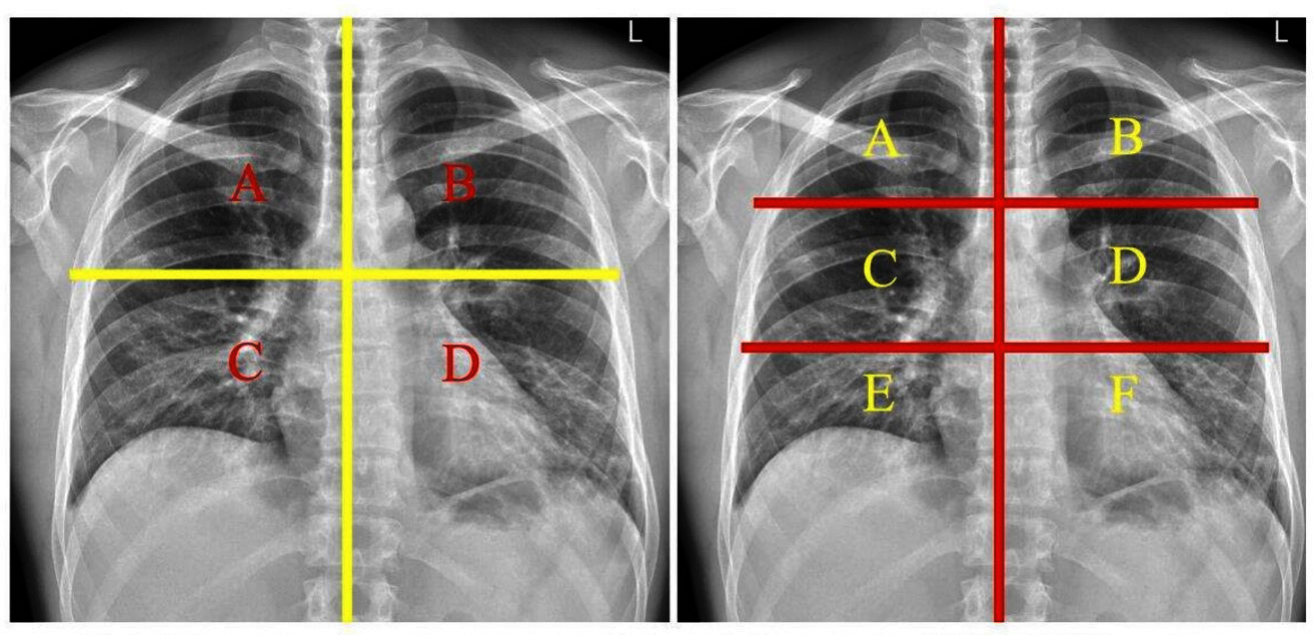
Chest quantitative techniques demonstrates the most common quantitative techniques used in COVID-19 CXR reporting.

Figure 3 Two examples of the quantitative four or six region assessments.

The anatomy is divided into four or six regions and each assigned a scale of usually 1–3 with increased disease the higher number. The regions are totalled to give a final infection score. The advantages of the quantitative method are its utility as a simple, quick, yet reliable tool and easily understood by any clinician.

## Discussion

This review has examined the use of templated radiological reporting tools in the assessment of the CXRs of COVID-19 patients. The qualitative structured reporting methods are recommended by most radiological societies. However, there were only three research studies found via an extensive web search that used a qualitative method, and these studies had variable findings. One study concluded that the use of the CXR to assess COVID-19 status was not statistically correlated with any clinical findings. However, a second found that the qualitative reporting method was a useful tool in the assessment of COVID-19 CXRs, but the authors also noted several limitations. The third study did show a positive conclusion where chest radiography of COVID-19 patients was predictive of a positive RT-PCR and suggested that the CXR could be useful in recognising undiagnosed COVID-19. The difference in the structured quantitative reporting tool used in this instance was designed by the authors themselves which offered more result categories within their reporting template compared to the recommended reporting tools from the various radiology societies.

This review has demonstrated that the quantitative structured reporting tools are preferred in studies within clinical environments. The reasons for this could be related to the value of a concise and uniform descriptive language of reporting by correlating disease to numerical scores. The quantitative method also appears to offer a good prognostic assessment of rates of disease along with the likelihood of future intubation and mortality risks. Judicious use of the tool should be considered as early diagnosis can be equivocal.

In searching and researching the use of structured reporting templates, we acknowledge that there was no comparison of this reporting method to that of free text radiological reporting. Free text style, also known as narrative reporting, is widely used in radiology reporting and has been an essential part of clinical communication in radiology discourse for over a century and will clearly continue to be used.^21^ We did not find any articles that compared the utility of structured templates to that of free text style, or indeed any articles that compared radiologist perceptions on the use of the structured reporting templates.

As COVID-19 infection and transmission rates in time decline and eventually COVID retreats from public discourse, it is of significant public health benefit that efficient and speedy diagnostic tools are in place to meet any future re-emerging of this contagion. The history of public health in the past 20 years is replete with life threatening contagions such as Ebola, swine flu, and MERS, each with significant mortality rates. For this reason, it is vital that we are pre-prepared as much as we reasonably can. It is the synergistic action of multiple health strategies, including radiology, that contain illness and hopefully an efficient diagnostic tool not unlike the templated quantitative reporting tool we have developed can play a part in any future return of SARS-CoV-2 or any other significant new respiratory virus.

## Summary

There is limited evidence for the implementation or utility for structured reporting templates, primarily due to the limited research scope where they have been deployed. There is however evidence demonstrated by this review that the quantitative structured style of reporting instrument features more prominently in research articles compared to the alternative qualitative design when reporting CXRs on patients with COVID-19, even though the qualitative structured design is advocated by most known international radiology societies.

At this point, it is speculative to determine why the quantitative style is preferred by researchers. It could be that a numerical score is an easier method of disease analysis within health environments where the immediacy of clinical results is a requisite within the persistent presence SARS-CoV-2. The answer could also be found in qualitative report designs where subjective terms such as indeterminate, high suspicion, characteristic or unlikely could be too equivocal for the referring clinician. Moreover, the comprehensive search of four databases did not find any studies that compared structured reporting quantitative to qualitative templates for COVID-19.

It is evident that more research is required to not only explore the future use of structured qualitative reporting, but to also examine studies that compare the utility of both reporting methods for COVID-19 CXRs or possibly combine elements of both quantitative and qualitative language within one structured format. The implementation of structured reporting templates also needs to be rigorously assessed for consistency, utility and clarity of reporting quality for all stakeholders including readers and referrers in the reporting of COVID-19 CXRs into the future.
